# Critical Review of Cataractogenesis and Lens Contouring in Radiation Oncology

**DOI:** 10.1016/j.adro.2026.102100

**Published:** 2026-05-22

**Authors:** Alexander Grosinger, Anna Roovers, Kyra M. Boorsma Bergerud, Marissa Kaufman, Jessica Vadas, Richard Evans, Gregory Hubert, Sara R. Alcorn, Jianling Yuan, Jessica Lawrence, Colette Shen, Collin M. McClelland, Michael S. Lee, Stephanie Terezakis, Lindsey Sloan

**Affiliations:** a Department of Radiation Oncology, University of Minnesota, Minneapolis, Minnesota; b University of Minnesota, Minneapolis, Minnesota; c University of Minnesota Medical School, Minneapolis, Minnesota; d Masonic Cancer Center, University of Minnesota, Minneapolis, Minnesota; e Department of Surgical & Radiological Sciences, University of California Davis, Davis, California; f Department of Radiation Oncology, University of North Carolina, Chapel Hill, North Carolina; g Department of Ophthalmology and Visual Neurosciences, University of Minnesota, Minneapolis, Minnesota

## Abstract

**Purpose:**

The objective is to comprehensively review radiation-induced cataractogenesis and recommend lens contouring guidelines to unify and improve dose reporting that informs future lens constraints.

**Methods and Materials:**

A thorough literature review was conducted to examine the anatomic, physiological, and pathologic aspects of the lens. Studies on radiation-induced cataracts, including thresholds for cataractogenesis, latency periods, and classification systems, were reviewed. Standardized lens contouring guidelines were developed for use in radiation therapy treatment planning and dose reporting.

**Results:**

The lens is a transparent, avascular structure responsible for refraction and accommodation. It is highly radiosensitive, and radiation-induced cataractogenesis most often presents as posterior subcapsular cataracts, although cortical and nuclear sclerotic changes can also occur. Key risk factors include total dose, fractionation, and dose rate. Cataracts are a well-described late effect after ocular-directed radiation therapy and other treatments that deliver clinically meaningful lens dose (eg, ocular radiation therapy/brachytherapy and total body irradiation), with incidence varying by lens dose, fractionation, patient age, and duration of follow-up. Standard assessment tools—slit-lamp examination and the Lens Opacities Classification System III—are limited by interobserver variability. Scheimpflug tomography offers a promising, objective, quantitative alternative. We present a step-by-step guide to improve consistency in lens delineation for radiation therapy planning, facilitate avoidance when feasible, and standardize lens dose reporting for future studies.

**Conclusions:**

Radiation-induced cataracts are a significant late complication of ocular, brain, total body, and head and neck radiation therapy. Standardized lens contouring guidelines and multidisciplinary collaboration with ophthalmology can improve lens-sparing and enhance detection and management of cataracts. Further research is needed to refine dose thresholds, improve assessment methods, and develop strategies to mitigate cataractogenesis.

## Introduction

Radiation-induced cataract formation is a clinically significant late effect of ocular, cranial, total body, and head and neck irradiation. A cataract is defined as an opacification of the normally transparent crystalline lens that results in impaired transmission of light to the retina and progressive visual dysfunction. Cataracts may develop through a variety of mechanisms, including aging, metabolic disease, corticosteroid exposure, trauma, and ionizing radiation.

Ionizing radiation is believed to induce cataractogenesis by damaging proliferating epithelial cells of the lens, particularly in the germinative zone.^1^^,^^2^ Radiation injury disrupts normal cellular differentiation and repair processes, leading to abnormal migration of damaged epithelial cells and progressive accumulation of aberrant lens fibers and protein aggregates.^3^ These structural changes ultimately result in increased light scattering and lens opacification. The biological processes underlying radiation-induced cataractogenesis contribute to the delayed onset of clinical cataracts, which may develop months to years after radiation exposure.^3^

The latency period for radiation-induced cataracts varies widely depending on dose, fractionation, and patient-related factors, but cataracts are commonly reported within 2 to 5 years after high-dose ocular irradiation, whereas lower dose exposures may demonstrate latency periods of 5 to 10 years or longer.^4^^,^^5^

Radiation-induced cataract formation is traditionally considered a deterministic tissue reaction, meaning that the probability and severity of the effect increase once a threshold dose is exceeded. The International Commission on Radiological Protection currently proposes a threshold dose of approximately 0.5 Gy for detectable lens opacities, although ongoing epidemiologic studies suggest that cataract formation may occur at lower doses.^6^, ^7^, ^8^

Objective monitoring of lens opacification has historically relied on slit-lamp examination and grading systems such as the Lens Opacities Classification System III.^3^ More recently, Scheimpflug tomography, a rotating camera imaging technique that captures cross-sectional images of the anterior segment of the eye, has emerged as a quantitative method for assessing lens density and detecting early lens opacities with improved reproducibility.^9^^,^^10^

## Methods and Materials

A literature search was conducted to identify studies evaluating radiation-induced cataractogenesis, lens radiosensitivity, and considerations relevant to lens contouring during radiation therapy planning. Electronic databases, including PubMed/MEDLINE, Embase, and Web of Science, were queried from database inception through September 2025. Search terms included combinations of the following keywords and Medical Subject Headings (MeSH): *radiation-induced cataract, radiation cataractogenesis, lens radiation dose, ocular radiation toxicity, radiotherapy lens toxicity, posterior subcapsular cataract, lens dose constraint*, and *lens contouring in radiotherapy*. Boolean operators (“AND” and “OR”) were used to combine search terms to optimize sensitivity.

**Figure 1:**
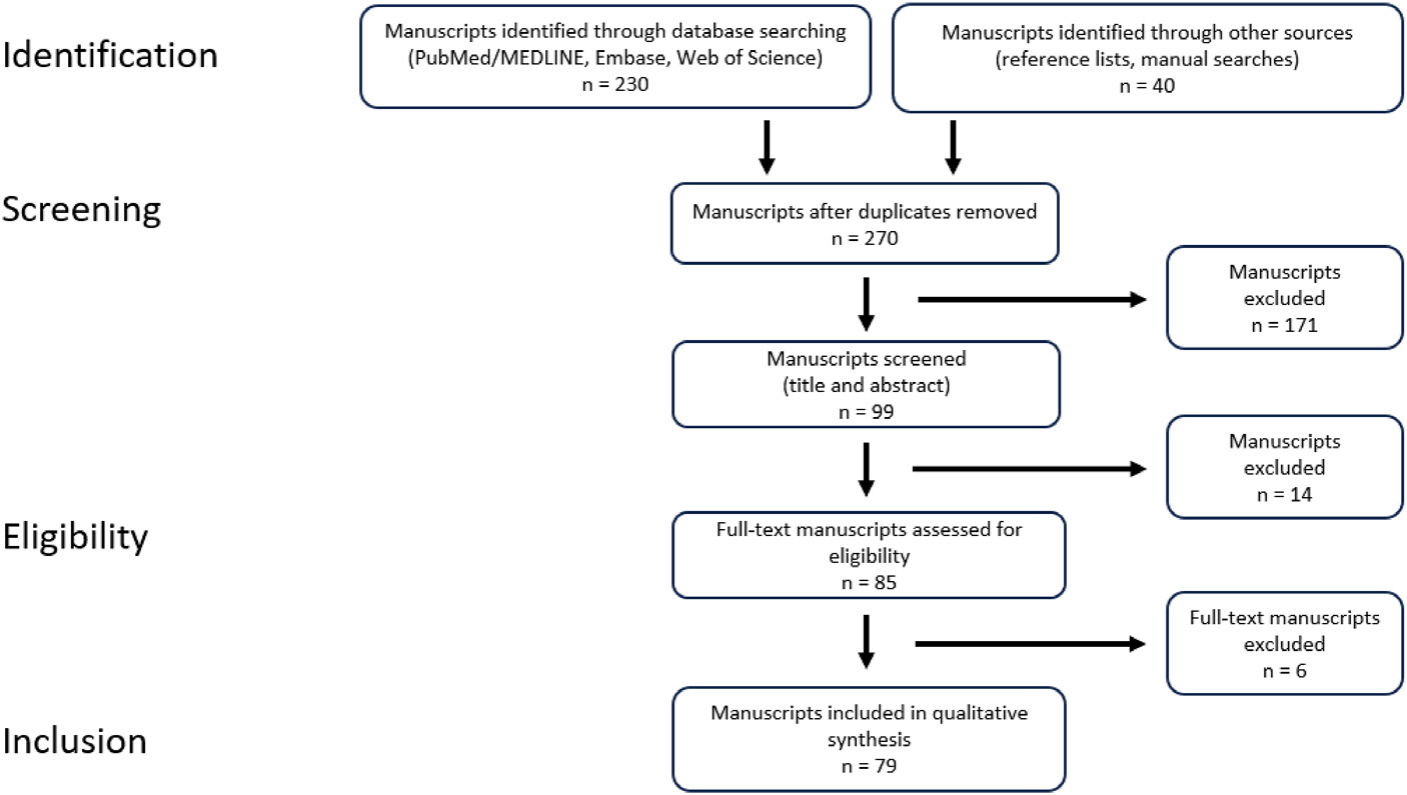
Preferred Reporting Items for Systematic reviews and Meta-Analyses (PRISMA) flow diagram of literature search and study selection.

Titles and abstracts were screened to identify studies addressing radiation exposure and cataract formation across environmental, occupational, diagnostic, and therapeutic contexts. Full-text articles were reviewed when abstracts suggested relevance to radiation-associated lens injury, cataract incidence, dose-response relationships, latency patterns, or clinical assessment methods. Reference lists of relevant publications and review articles were also examined to identify additional studies. Only English-language publications involving human subjects were included. Case reports, editorials, and studies that lacked sufficient information on radiation exposure or cataract outcomes were excluded. Relevant studies were grouped by exposure context, including environmental or nuclear exposure, occupational radiation exposure, diagnostic radiation exposure, and therapeutic radiation exposure, which informed the synthesis summarized in the manuscript tables. The study selection process is summarized in a Preferred Reporting Items for Systematic reviews and Meta-Analyses flow diagram (Fig. 1).

### Anatomy of the lens

**Figure 2:**
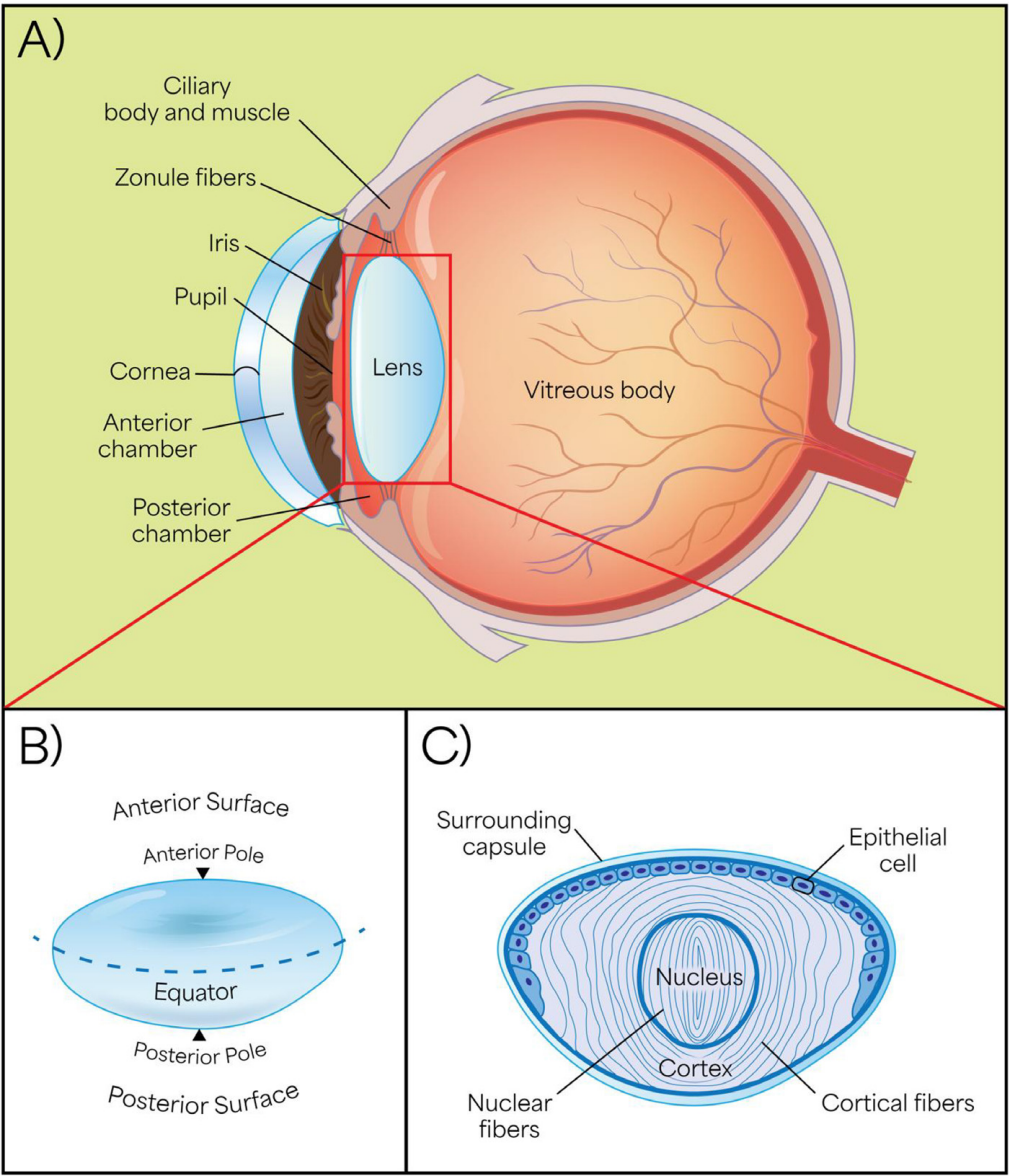
Anatomy of the anterior chamber and lens. The lens is a transparent, biconvex, avascular structure in the anterior eye that is positioned between the iris and vitreous cavity. It is encased in an elastic, collagenous capsule that is thickest at the equator and thinnest at the posterior pole. Zonular fibers suspend the lens from the ciliary body. (B) Three-dimensional appearance of the lens. (C) Cross-section through the lens showing a single layer of cuboidal subepithelial cells peripherally, the mid-peripheral cortex, and the central nucleus.

The lens is a transparent, ellipsoid, biconvex, avascular, and denervated crystalline structure located in the anterior segment of the eye, posterior to the iris and anterior to the vitreous cavity.^11^ The anterior surface of the lens is less convex than the posterior, and both surfaces meet at the lens equator (Fig. 2).^1^

Surrounding the lens is the lens capsule, an elastic, transparent, and collagenous basement membrane.^11^ The capsule is thickest at the equator and thinnest at the posterior pole.^1^ Zonular fibers connect and suspend the lens capsule from the equator to the ciliary body.^12^ Beneath the capsule lies the lens epithelium, composed of a single layer of cuboidal epithelial cells.^1^ The germinative zone, just anterior to the equator, is the most mitotically active region of the lens epithelium.^1^^,^^2^

The bulk of the lens comprises fibers derived from the lens epithelium, which possess a long, crescent shape and hexagonal cross-section.^1^^,^^12^ These fibers are arranged in concentric layers, resembling an onion.^12^ They do not divide and persist throughout life, but new fiber formation continuously pushes older fibers toward the center, where they overlap at structures called sutures.^1^^,^^11^ During cellular differentiation, intracellular organelles degrade, whereas cell membrane density increases to reduce light scattering.^13^ Crystallins are the predominant lens protein, preventing partially denatured proteins from aggregation and maintaining transparency.^2^ Lens fibers are tightly packed and highly ordered, forming layers that include, from the innermost to outermost, the nucleus, cortex, and capsule.^1^

### Physiology of the lens

Along with the cornea, the lens transmits, refracts, and focuses incoming light onto the retina.^2^ Unlike the fixed cornea, the lens alters its refractive power, allowing vision at varying distances through accommodation.^13^ Accommodation is mediated by the ciliary muscles, which attach to zonular fibers.^13^ Relaxation of the ciliary muscles increases tension on the zonular fibers, flattening the lens for distance vision.^13^ Conversely, ciliary muscle contraction relieves tension, allowing the lens to thicken and curve for near vision.^13^ With age, the lens stiffens and reduces accommodative ability in a process termed presbyopia.^1^

Lens transparency is essential for proper function and is maintained through its highly ordered architecture, which minimizes light scattering and provides a refractive gradient to correct spherical aberration.^1^^,^^2^^,^^13^ The lens also protects the retina from ultraviolet light exposure.^11^ Because the lens is avascular and lacks nerves, it relies on glucose metabolism for energy.^1^^,^^13^ Selective permeability of the lens capsule regulates molecular passage, ensuring homeostasis.^13^ Disruptions in enzymatic activity or electrolyte balance can lead to metabolic disturbances, protein denaturation, and cataract formation.^1^

**Figure 3:**
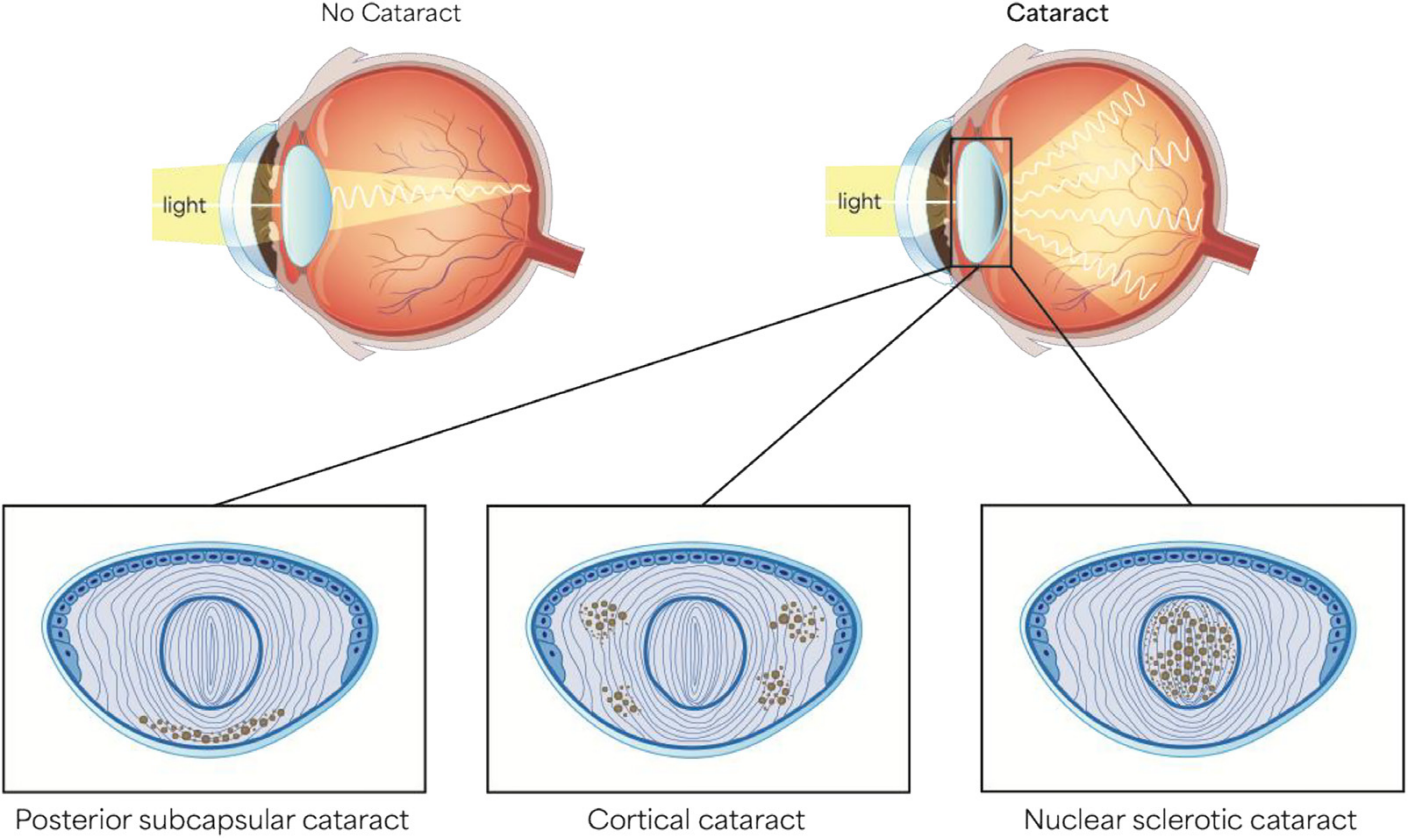
Different cataract formations. The lens and cornea transmit, refract, and focus light onto the retina. When light strikes the cataract, it may scatter the light not allowing it to focus. The primary cataract types include posterior subcapsular (forming along the posterior capsule), cortical (radial opacities in the cortex), and nuclear sclerotic (progressive nucleus sclerosis and yellowing). Posterior subcapsular cataracts are more often associated with radiation exposure than cortical or nuclear sclerotic cataracts.

Cataracts are classified based on their anatomic location within the lens (Fig. 3).^3^ Cataracts generally result in symptoms of glare, reduced contrast sensitivity, color desaturation, and difficulty with near or far vision, depending on their location.^3^ The 3 most common cataract types are posterior subcapsular, cortical, and nuclear sclerotic.^3^ Posterior subcapsular cataracts develop in the posterior aspect of the lens, just beneath the capsule, and are commonly associated with radiation exposure, prolonged corticosteroid use, intraocular inflammation, and systemic conditions such as diabetes mellitus.^3^ Cortical cataracts originate in the lens cortex and are characterized by radial or wedge-shaped opacities that extend toward the nucleus of the lens and are frequently associated with aging, metabolic disorders, and oxidative stress.^3^ Nuclear sclerotic cataracts involve progressive sclerosis and yellowing of the lens nucleus due to protein aggregation and oxidative damage, and are predominantly age related.^3^

### Cataractogenesis secondary to ionizing radiation

The lens is highly radiosensitive, and radiation exposure is a well-described cause of cataractogenesis.^9^^,^^14^ Early studies of atomic bomb survivors identified posterior subcapsular cataracts as the most common radiation-induced subtype, although cortical and nuclear cataracts were also observed.^15^, ^16^, ^17^, ^18^, ^19^, ^20^ Similar findings were reported in survivors of the Chernobyl disaster, where a large population was exposed to low-dose radiation.^21^, ^22^, ^23^, ^24^ A comprehensive list of studies evaluating environmental and accidental exposures resulting in cataract development is summarized in Table 1.

**Table 1:** Studies evaluating cataractogenesis secondary to nuclear accidents and environmental exposures

Patient population	Absorbed or equivalent dose	Absorbed dose (Gy)	Clinical findings	Reference
Populations exposed to low-dose radiation in Yangjiang, China	0.068-0.31 Gy	0.068-0.31	Cumulative radiation dose was significantly associated with increased risk for cortical and PSC opacities. The threshold dose for cortical lens opacity was estimated to be 0.14 Gy. No statistically significant threshold was obtained for PSC lens opacity.	Su et al^24^
Chernobyl liquidators	0.033 ± 0.003- 0.182 ± 0.006 Gy	0.033 ± 0.003-0.182 ± 0.006	PSC opacity was primarily observed in the group exposed to highest dose (0.182 ± 0.006 Gy).	Yeltokova et al^75^
Chernobyl clean-up workers	0.0-0.5 Gy	0.0-0.5	The risk for cataract development was significantly elevated at 1 Gy. Early-stage PSC changes and PSC cataract formation followed a statistically significant dose-response trend.	Worgul et al^22^
Atomic bomb survivors	0.0-1.0 Gy	0.0-1.0	Higher doses were associated with increased prevalence of post-exposure cataracts.	Neriishi et al^20^
Atomic bomb survivors	0.0-4.94 Sv	0-4.94	RT dose was significantly correlated with the prevalence of cortical opacities and PSC opacities.	Nakashima et al^16^
Atomic bomb survivors	<0.005-2.0 Sv	<0.005-2.0	The prevalence of cortical and PSC opacities was significantly associated with increased radiation dose.	Minamoto et al^15^
Populations exposed to chronic low-dose γ radiation in Taiwan	0.001-1.493 Sv	0.001-1.493	A significant association between dose and lens opacification was found in populations exposed to radiation before age 20.	Chen et al^23^
Populations near Chernobyl, ages 5-17 at the time of exposure	0.0-0.086 Sv	0.0-0.086	PSC opacification was significantly more frequent in exposed vs unexposed children. The frequency of cortical opacification in exposed vs unexposed participants was not statistically different.	Day et al^21^
Atomic bomb survivors	<0.01-2.00+ Gy	<0.01-2.00+	Radiation dose was associated with increased prevalence of axial opacities, except in the oldest age group (>70 y). Radiation dose was associated with increased prevalence of PSC opacities in all age groups.	Otake et al^19^

*Abbreviations*: PSC = posterior subcapsular; RT = radiation therapy.

Reported lens dose reflects the exposure values as presented in the original publications, which may include different radiation units (eg, Sv, mSv, and Gy) or dose ranges. Standardized dose (Gy) represents the corresponding absorbed dose converted to Gray when possible to facilitate comparison across studies.

There is no global consensus on the threshold radiation doses for cataract formation. The International Commission on Radiological Protection (ICRP) proposes a threshold dose of 0.5 Gy, but some studies suggest a lower threshold.^6^, ^7^, ^8^ Occupational radiation exposure has been associated with posterior subcapsular cataracts in health care workers, airline pilots, and astronauts, although findings are inconsistent.^25^, ^26^, ^27^, ^28^, ^29^, ^30^, ^31^, ^32^, ^33^, ^34^, ^35^, ^36^, ^37^, ^38^, ^39^, ^40^, ^41^ Similarly, the relationship between diagnostic computed tomography (CT) exposure and cataract risk remains unclear due to conflicting studies.^42^, ^43^, ^44^ Discordant studies highlighting the presence or absence of a statistical association between occupational exposures and cataractogenesis are summarized in Table 2.

**Table 2:** Studies evaluating cataractogenesis secondary to occupational exposures

Patient population	Reported lens dose	Standardized dose (Gy)	Clinical findings	Reference
Interventional cardiologists	0.224 Sv (left lens); 0.085 Sv (right lens)	0.224; 0.085	No statistically significant differences in nuclear, cortical, and PSC opacities were found in exposed vs unexposed participants.	Alhasan and Aalam^32^
Interventionalists	17.1 Sv	-	PSC and cortical cataract prevalence were increased in exposed vs unexposed participants, but the difference was not statistically significant.	Rose et al^36^
Interventional cardiology personnel	Not reported	-	No difference in the frequency of cortical cataracts was found between exposed vs unexposed populations. PSC cataract was significantly more prevalent in exposed vs unexposed participants.	Barbosa et al^38^
Interventionalists	Not reported	-	Nuclear opacities were observed more frequently in exposed vs non-exposed participants.	Scheidemann-Wesp et al^26^
Mayak production association workers	0.0-2.0+ Sv	0.0-2.0+	Higher dose exposure increased the risk of cataract removal, but this trend was not statistically significant.	Azizova et al^39^
Interventional cardiologists and catheterization laboratory staff	0.1-22.5 Gy	0.1-22.5	Cortical and PSC opacity changes were more prevalent in exposed participants than in unexposed participants. Radiation exposure was independently associated with cortical and PSC opacity changes. No significant association between cumulative lens dose and changes to the lens was found.	Karatasakis et al^25^
Radiologic technologists	<0.01-0.5+ Gy	0.01-0.5	Significant risk for cataract prevalence was found for the full range of cumulative doses, including persons with a cumulative dose under 100 mGy.	Little et al^30^
Interventional cardiology personnel	0.006 ± 0.006- 0.017 ± 0.012 Sv	0.006 ± 0.006-0.017 ± 0.012	PSC opacity findings were more common in interventional cardiologists (mean of 9.1 ± 9.5 mSv) compared to technicians (mean of 5.6 ± 6.8 mSv). PSC lens changes were correlated with increased duration of work experience and the number of procedures conducted.	Bitarafan Rajabi et al^27^
Physicians exposed to occupational radiation	0.072 Sv (mean)	0.072	No significant increase in cortical or PSC opacities was observed in exposed vs unexposed participants.	Auvinen et al^31^
Interventional cardiologists	Not reported	-	PSC opacification was significantly more likely in interventional cardiologists exposed to radiation than in unexposed participants.	Jacob et al^37^
Interventional catheterization professionals	1.0-4.7 Sv	1-4.7	Increased radiation exposure was significantly associated with PSC opacification.	Vano et al, 2013^33^
Interventional cardiology personnel	0.0-6.0 Sv	0-6.0	Cardiologists exposed to radiation (6.0 Sv mean) had a significantly higher prevalence of lens opacities and a 3-fold higher risk of PSC opacification than unexposed participants. Nurses and technicians exposed to radiation (1.5 Sv mean) had a higher, but non-significant prevalence of PSC opacities than unexposed participants.	Vano et al^34^
Interventional cardiology personnel	0.01-43.0 Gy	0.01-43.0	The prevalence of PSC opacities was significantly higher in interventional cardiologists (mean of 3.7 Gy) and nurses (mean of 1.8 Gy) compared with unexposed controls. In interventional cardiologists, PSC opacity changes were correlated with cumulative dose.	Ciraj-Bjelac et al^35^
Astronauts	0.015-0.129 Sv	0.015-0.129	Astronauts exposed to radiation had more extensive cortical and PSC opacification than unexposed astronauts. Increased dose exposure was associated with higher PSC opacity but not cortical opacity.	Chylack et al^29^
Radiologic technologists	0.005-0.06 Gy (lowest group mean – highest group mean)	0.005-0.06	Occupational exposure to radiation (mean 28.1 mGy) increased the risk of cataract (ERR = 1.98). PSC lens opacities were the most observed changes. The highest dose group (mean 60.1 mGy) had a hazard ratio of 1.18 compared to the lowest dose group (mean 5.1 mGy).	Chodick et al^28^
Commercial airline pilots	0.001-0.048 Sv	0.001-0.048	Cumulative radiation dose was significantly associated with risk for nuclear cataracts. No significant association between cumulative dose and risk for cortical or PSC cataracts was found.	Rafnsson et al^7^
Astronauts and cosmonauts	Not reported	-	The average PSC opacity was higher in astronauts and cosmonauts exposed to radiation than in unexposed jet pilots.	Rastegar et al^40^
Astronauts	0.0-0.091 Sv	0.0-0.091	The high-dose group (>8 mSv) had a significantly higher risk of cataract development than the low-dose group (<8 mSv). In the high-dose group, significant hazard ratios for PSC, nuclear, or mixed cataracts were found, while no significance was found for cortical or dot opacities. Protective eyewear minimizes risk.	Cucinotta et al^8^

*Abbreviations*: CT = computed tomography; ERR = excessive relative risk; PSC = posterior subcapsular; RT = radiation therapy.

Reported lens dose reflects the exposure values as presented in the original publications, which may include different radiation units (eg, Sv, mSv, Gy) or dose ranges. Standardized dose (Gy) represents the corresponding absorbed dose converted to Gray when possible to facilitate comparison across studies.

Monitoring protocols for objective lens changes after irradiation, including cataract development, are most developed after ocular-directed oncologic treatments, such as those for uveal melanoma, because of the potential for rare, irreversible optic toxicities.^9^ The Common Terminology Criteria for Adverse Events classification system is widely used to document treatment-related toxicities and specifically accounts for cataractogenesis.^9^ Formal clinical vision assessments for all patients at risk, however, are infrequently performed during radiation therapy.^9^ Differentiating radiation-induced cataracts from those due to aging, trauma, or corticosteroid use poses challenges.^9^ This has led to the concept of a “cataractogenic load,” referring to a radiation-induced acceleration of natural age-related cataractogenesis.^45^, ^46^, ^47^

Prior studies have attempted to correlate cataract development with therapeutic radiation exposure. Mathis et al^46^ used the Lens Opacities Classification System III to characterize radiation-induced cataracts after proton beam therapy.^3^ However, accurate classification requires extensive training and remains subject to interobserver variability.^3^^,^^46^ Latency between radiation exposure and lens opacification is influenced by dose rate, fractionation, corticosteroid use after total body irradiation, and total radiation dose.^4^^,^^5^ Fukutsu et al^48^ reported that 52.3% of eyes in patients with ocular adnexal mucosa-associated lymphoid tissue lymphoma required cataract surgery within an average of 43 months after radiation therapy.

Although cataract formation represents a potential late toxicity of radiation therapy, cataracts remain highly treatable. When cataracts become visually significant, the standard treatment is cataract extraction, most commonly performed via phacoemulsification with intraocular lens implantation, which generally results in meaningful improvement in visual acuity. In postradiation settings, surgical planning may require additional attention to coexisting ocular morbidity (eg, radiation retinopathy or maculopathy), but reported series suggest that cataract surgery remains feasible and effective for many patients with radiation-associated cataracts.^49^

Cataractogenesis in pediatric populations follows a similar trend. Nguyen et al^50^ demonstrated that, among children treated for retinoblastoma, whole-eye radiation therapy resulted in cataract development in 71.1% of eyes, compared with 35.3% in those receiving lens-sparing radiation therapy, with an average latency of 51.8 months. The US Childhood Cancer Survivor Study reported a 5-year cataract prevalence ranging from 0.9% to 3.8%, depending on cancer histology.^51^ Allodji et al^52^ estimated a 2.3% cataract incidence in children treated with radiation therapy for primary central nervous system tumors. Among pediatric patients receiving total body irradiation of 6 Gy to 12 Gy, 50% to 90% developed cataracts within 10 years.^53^ Van Kempen-Harteveld et al^54^ further demonstrated a 3-fold increase in cataract risk among patients without versus with eye shielding treated with total body irradiation. Patterns of cataractogenesis in childhood cancer survivors remain elusive, as most studies included non-eye-shielded patients and short follow-up durations of less than 10 years.^6^^,^^53^, ^54^, ^55^, ^56^

A 5-year cataract incidence of 20% to 25% has been associated with a mean lens dose of 7 Gy.^57^^,^^58^ Higher doses correlate with a shorter latency period.^57^^,^^58^ Mathis et al^46^ found that in proton therapy patients, posterior subcapsular cataract severity correlated with dose to the lens, peripheral lens dose, and ciliary body. Hypofractionated, high-dose proton treatment resulted in 15% of patients developing cataracts, with 9% requiring surgical correction within a median of 1.6 to 2.3 years.^45^ Conventional radiation therapy, in contrast, is associated with cataract detection 5 to 10 years after treatment.^9^ The long latency, combined with intermittent and nonstandardized ophthalmology re-evaluations, complicates establishing causality. Importantly, the risk of cataract development is likely underestimated due to the limited follow-up durations in published literature.^9^

Ocular brachytherapy routinely delivers doses that exceed ICRP thresholds.^59^ Puusaari et al^60^ reported a hazard ratio of 1.15 per 10-Gy increase in patients undergoing ocular brachytherapy. Finger et al^61^^,^^62^ found a 5-year cataract incidence of up to 83% in patients who completed brachytherapy, with surgical correction required in 12% of patients. Studies reporting cataract development after radiation treatment are summarized in Table 3.

**Table 3:** Studies evaluating treatment-related and diagnostic-related cataractogenesis

Patient population	Reported lens dose	Standardized dose (Gy)	Clinical findings	Reference
Head CT patients	0.0-0.633 Gy	0.0-0.633	The minimum value for a significant increase in cataract risk in a 3-y lagged model was 100.0 mGy and 250.0 mGy for 5 and 7-y lagged models.	Emami et al^43^
Head and neck radiation therapy patients	0.01-20.0 Gy (left lens); 0.01-22.93 Gy (right lens)	0.01-20.0; 0.01-22.93	Changes in lens opacity could be seen 3 mo after radiation therapy completion. Opacity presence followed a dose-responsive trend at 3 mo and 6 mo postradiation therapy.	Arefpour et al^76^
Head CT patients	0.005-0.1 Gy	0.005-0.1	1-3 CT scans increased the risk of cataract surgery by 3%-8%; however, subsequent CT scans showed nonstatistically significant decreases in cataract risk. Low-dose radiation from CT scans was not associated with increased risk for cataract surgery.	Gaudreau et al^44^
Retinoblastoma patients receiving lens-sparing RT or whole-eye RT	9.93 Gy (median for lens-sparing RT); 37.53 Gy (median for whole-eye RT)	9.93; 37.53	Increased lens dose was significantly associated with higher cataract hazard, and the mean lens dose was a significant predictor of cataract incidence within 5 y.	Nguyen et al^50^
Childhood cancer survivors	2.6 Gy (left mean); 2.5 Gy (right mean)	2.6; 2.5	Patients receiving RT had a 4.4-fold higher hazard for cataracts. Radiation exposure to both eyes was associated with a significant risk of cataracts.	Allodji et al^52^
Childhood cancer survivors	0.0-66.0 Gy	0-66.0	Higher lens dose was associated with increased prevalence of cataracts. Elevated risk for developing cataracts was observed at doses >0.5 Gy.	Chodick et al^51^
Adult retinoblastoma survivors	0.3-22.0 Gy	0.3-22.0	Cataract extraction was significantly associated with the number of courses of external radiation treatment. Cataract extraction was 6-fold higher at doses of 5-22.0 Gy compared with doses below 2.5 Gy.	Chodick et al^56^
Hemangioma radiation therapy patients <age 18	0.0-8.4 Gy	0-8.4	Higher dose was significantly associated with increased risk of PSC and cortical opacities but not nuclear opacities.	Hall et al^77^
Skin hemangioma radiation therapy patients analyzed 30-45 y after exposure	1.1-8.4 Gy	1.1-8.4	A high prevalence of PSC opacities was observed in adults exposed to radiation therapy in infancy, most prominently at doses above 2.0 Gy. Higher cataract grade correlated with increased radiation dose.	Wilde et al^78^
Total body irradiation patients	<0.048-≥0.09 Gy/min (instantaneous dose rate)	<0.048-≥0.09	Risk of cataract incidence after 5 y was significantly increased in both the high instantaneous dose group (≥0.09 Gy/min) and the medium dose group (<0.09 Gy/min) in comparison to the low-dose group (<0.048 Gy/min).	Belkacémi et al^79^
Diagnostic x-ray exposure	Not reported	-	History of CT scan was significantly associated with the presence of cortical and PSC opacities.	Klein et al^42^

*Abbreviations*: CT = computed tomography; ERR = excessive relative risk; PSC = posterior subcapsular; RT = radiation therapy.

Reported lens dose reflects the exposure values as presented in the original publications, which may include different radiation units (eg, Sv, mSv, Gy) or dose ranges. Standardized dose (Gy) represents the corresponding absorbed dose converted to Gray when possible to facilitate comparison across studies.

### Improving cataract detection and risk factors for development in radiation therapy patients

Serial, complete ophthalmic examinations are critical to clarify temporal, anatomic, and dose- and delivery-related associations between irradiation and cataract development. Slit-lamp examinations are commonly used for the subjective evaluation of cataract formation and location; however, significant interobserver variability may lead to inaccurate assessments.^9^ In contrast, Scheimpflug tomography—an imaging technique that uses a rotating camera and slit-beam illumination to generate cross-sectional images of the anterior segment—provides an objective map of the lens, enabling quantitative identification of focal opacifications and improving the reproducibility of cataract detection with reduced examiner subjectivity.^9^ Eter et al^10^ pioneered its use in detecting lens opacities after radiation therapy. The integration of objective monitoring with Scheimpflug tomography offers an elegant solution for capturing key lens data after irradiation, with image generation that can be stored and retrospectively examined within large data sets.

Collectively, the published literature supports that lens opacification occurs following radiation doses that are lower than doses routinely delivered to the lens in patients treated for orbito-ocular, brain, and head and neck tumors. Cataract severity and frequency correlate with total radiation dose, fractionation, dose rate, and potentially radiation quality. The latency period and dose-response relationships require additional study to enable clear consensus guidelines on threshold dose, dose constraint recommendations, and optimal monitoring regimens.

Two primary recommendations will improve radiation reporting and detection of cataracts after treatment. First, establishing standard guidelines for lens contouring is vital to optimize conformal avoidance of accurately contoured sensitive structures and to reduce variability within and between radiation therapy departments.^63^ Reducing variability in lens contouring will improve comparisons across published literature and enable pooling of data for robust analysis of lens dose distributions and opacification patterns. The creation of a planning risk volume (PRV) to account for lens movement during radiation therapy may also be warranted, but may require institutional assessment of immobilization techniques and patient positioning.^64^^,^^65^ Several studies have demonstrated interfraction and intrafraction lens motion occurring within 2 mm of neutral gaze during radiation therapy.^66^^,^^67^ Second, incorporating ophthalmology within multidisciplinary care teams is critical for early detection, addressing patient management concerns, and answering lingering questions regarding radiation-induced cataracts. Prospective studies that include standardized ophthalmologic examinations before, during, and after radiation treatment are warranted to refine risk estimates and improve clinical evaluation strategies.

### Eye positioning, immobilization, and implications for lens dose

Accurate lens dose estimation relies on consistent contouring and reproducible eye positioning during both simulation and treatment. In ocular radiation therapy—particularly for uveal melanoma treated with proton therapy or stereotactic techniques—centers commonly employ gaze-control strategies such as neutral gaze or fixation-light guidance during simulation and treatment, sometimes supplemented with optical monitoring or tracking systems to improve the reproducibility of eye position.^68^^,^^69^ These approaches aim to reduce uncertainty in the relative positions of ocular structures and may improve the reliability of lens dose reporting and toxicity assessment. Given published evidence that ocular structures can move during treatment, it is important to consider immobilization techniques, gaze control, and CT and magnetic resonance imaging (MRI) slice thickness when interpreting lens dose metrics across studies and when deciding whether a lens PRV is appropriate.^64^, ^65^, ^66^, ^67^

### Contouring guidelines

**Figure 4:**
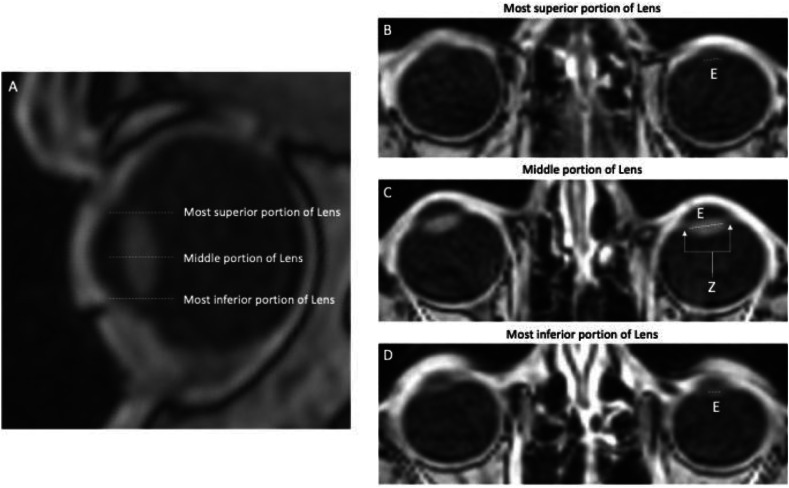
T1-weighted magnetic resonance imaging of the globe. Midline sagittal view (A) of the lens with corresponding axial sections (B-D) illustrates the lens anatomy. The superior, middle, and inferior portions of the lens in (A) correspond to axial sections (B), (C), and (D), respectively. The equator of the lens (E) and zonular fibers (Z) are labeled.

**Figure 5:**
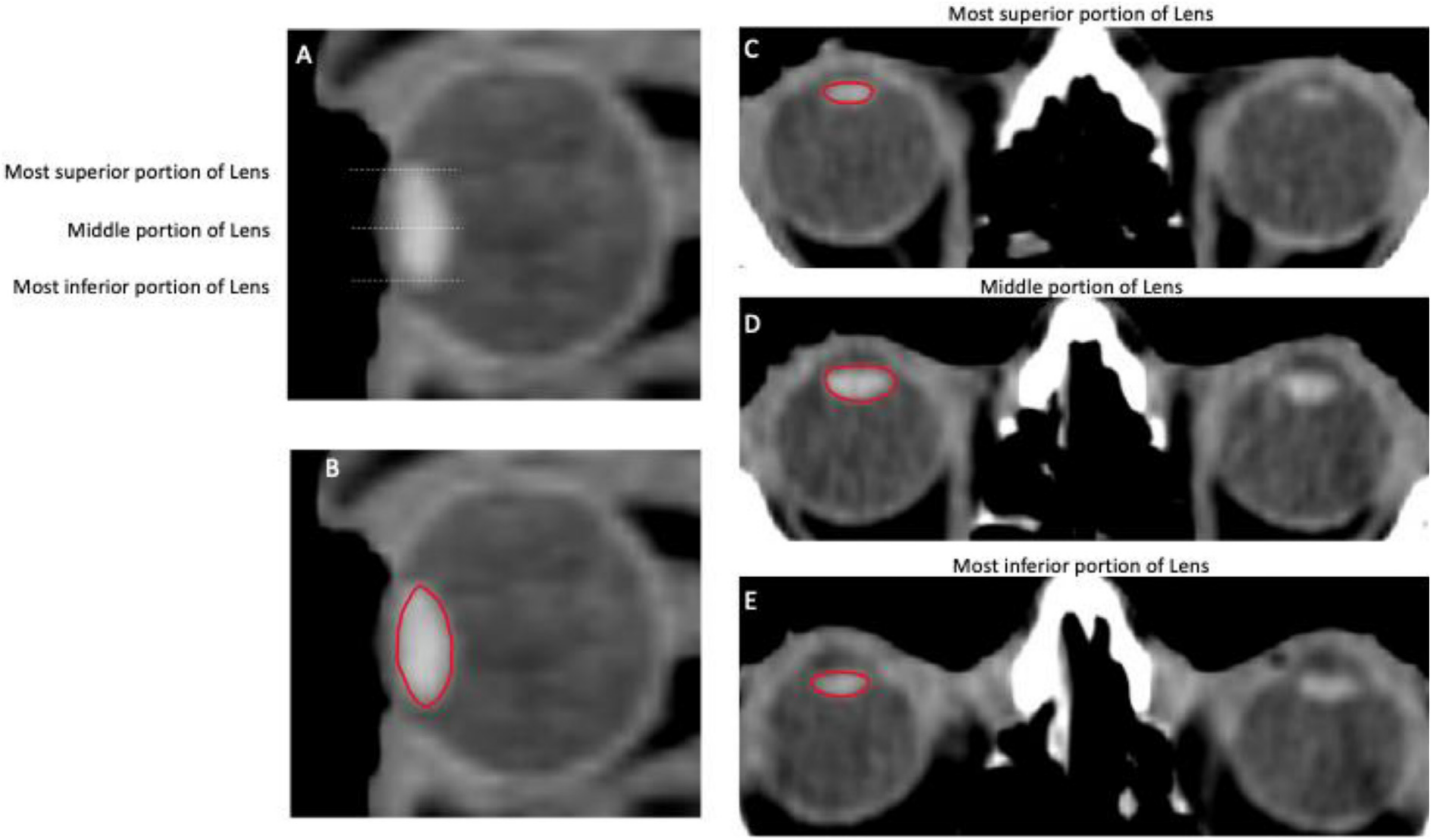
Computed tomography imaging of the globe for lens delineation. Midline sagittal view (A) and corresponding axial sections (C-E). The most superior portion of the lens in (A) aligns with axial section (C), the middle portion with (D), and the most inferior portion with (E). The red outline delineates the lens contour. Image (B) is identical to (A) but highlights the lens contour on the sagittal plane.

MRI correlates are helpful for identifying structures and anatomic landmarks (Fig. 4); thus, registering MRI to CT simulation images is recommended.^70^, ^71^, ^72^ The lens is to be contoured on the planning CT (Fig. 5). The adult human crystalline lens typically measures approximately 9 to 10 mm in equatorial diameter and 4 to 5 mm in anteroposterior thickness, although dimensions may vary slightly with age and accommodative state.^73^ These measurements may provide useful anatomic reference points during contouring to ensure that the delineated volume accurately reflects the expected lens dimensions.

Depending on patient positioning, CT simulation parameters, and slice thickness, the lens is typically visible through 3 to 6 axial slices during contouring. Thin-slice acquisitions (eg, 2 mm) may demonstrate a greater number of slices containing lens tissue, whereas thicker slice reconstructions (eg, 3 mm) may result in fewer slices capturing the full lens extent.^74^

Step 1: To begin contouring systematically, locate the middle of the native lens. Cross-reference this location using the sagittal and axial images to identify the anterior and posterior poles of the lens. The lens sits inside zonule fibers that radially connect to the lens capsule (Fig. 4), which are difficult to visualize on CT. The lens is anterior to the vitreous cavity and posterior to, yet abuts, the anterior segment. The lens is hyperintense to vitreous on T1-weighted MRI (Fig. 4) and hyperdense to vitreous on CT (Fig. 5).

Step 2: Tracking superiorly, the most cranial aspect of the lens will be the superior-most visible slice. The lens volume will follow the lens equator and extend to the anterior and posterior poles of the lens.

Step 3: Tracking inferiorly, the most caudal aspect of the lens will be the inferior-most visible slice. The lens volume will follow the lens equator and extend to the anterior and posterior poles of the lens.

Step 4: Cross-referencing the lens contour with a sagittal image is a useful final step to verify that the contoured edges are accurate. On sagittal imaging, the lens will appear lenticular, with an exaggerated convexity on the posterior pole compared with the anterior pole.

Step 5: Consider expanding by 2 mm, isotropically, to create a PRV for each lens when deemed appropriate through provider discretion. Consideration of immobilization approach and gaze control at simulation and treatment may inform whether a lens PRV is appropriate.

## Discussion

The lens is a highly radiosensitive structure, and exposure to ionizing radiation is a well-documented cause of posterior subcapsular, cortical, and nuclear cataracts. Despite this established risk, there is currently no global consensus on the exact threshold radiation dose for cataractogenesis. Although the ICRP proposes a threshold of 0.5 Gy, several studies suggest that the threshold may be even lower.^6^, ^7^, ^8^ Establishing definitive dose-response relationships and latency periods remains challenging due to limited follow-up durations in published studies and inconsistent, nonstandardized ophthalmologic evaluations. A primary challenge in current practice is the reliance on subjective monitoring techniques, such as slit-lamp examinations, which are prone to significant interobserver variability. To mitigate this, transitioning to objective imaging techniques, such as Scheimpflug tomography, is recommended. Because it provides a quantitative, reproducible map of the lens and reduces examiner subjectivity, Scheimpflug tomography may be used in future retrospective analyses of large data sets. Fostering the routine integration of ophthalmology into multidisciplinary oncology care teams is essential to facilitate standardized ophthalmic examinations before, during, and after radiation therapy.

Standardizing lens contouring is vital to optimizing conformal avoidance and reducing variability across radiation therapy departments. Accurate dose estimation relies heavily on consistent contouring practices, such as registering T1-weighted MRI to planning CT scans and leveraging the lens's distinct hyperintense and hyperdense appearance on these modalities. Finally, because the lens can experience interfractional and intrafractional motion, providers should carefully evaluate immobilization techniques and gaze-control strategies. When deemed appropriate, creating a 2 mm isotropic PRV expansion around the lens can account for lens movement, ensuring more reliable lens dose reporting and improved long-term visual outcomes.

Improving consistency in lens contouring during radiation therapy planning is essential for refining dose reporting, enabling more meaningful comparisons across studies, and ultimately supporting the development of evidence-based dose constraints. Through this narrative review and systematic approach to lens contouring, we aim to provide a reference on which to begin building this evidence.

## Conclusion

Future prospective studies incorporating standardized contouring, dose reporting, and ophthalmologic follow-up are needed to better characterize dose-response relationships and inform strategies to minimize radiation-associated cataractogenesis.

## Disclosures

Lindsey Sloan receives research and travel support from GT Medical Technologies. All other authors declare they have no competing interests.

## References

[1] Chen W TX, Chen X., Liu Y. (2017). Pediatric Lens Diseases.

[2] Hejtmancik J.F., Shiels A. (2015). Overview of the lens. Prog Mol Biol Transl Sci.

[3] Chylack L.T., Wolfe J.K., Singer D.M. (1993). The lens opacities classification system III. The longitudinal study of cataract study group. Arch Ophthalmol.

[4] Belkacémi Y., Touboul E., Méric J.B., Rat P., Warnet J.M. (2001). Radiation-induced cataract: Physiopathologic, radiobiologic and clinical aspects. Cancer Radiother.

[5] van Kempen-Harteveld M.L., Struikmans H., Kal H.B. (2000). Cataract-free interval and severity of cataract after total body irradiation and bone marrow transplantation: Influence of treatment parameters. Int J Radiat Oncol Biol Phys.

[6] Stewart F.A., Akleyev A.V., Authors on behalf of ICRP (2012). ICRP publication 118: ICRP statement on tissue reactions and early and late effects of radiation in normal tissues and organs–threshold doses for tissue reactions in a radiation protection context. Ann ICRP.

[7] Rafnsson V., Olafsdottir E., Hrafnkelsson J., Sasaki H., Arnarsson A., Jonasson F. (2005). Cosmic radiation increases the risk of nuclear cataract in airline pilots: A population-based case-control study. Arch Ophthalmol.

[8] Cucinotta F.A., Manuel F.K., Jones J. (2001). Space radiation and cataracts in astronauts. Radiat Res.

[9] Ainsbury E.A., Dalke C., Hamada N. (2021). Radiation-induced lens opacities: Epidemiological, clinical and experimental evidence, methodological issues, research gaps and strategy. Environ Int.

[10] Eter N., Wegener A., Schüller H., Spitznas M. (2000). Radiotherapy for age related macular degeneration causes transient lens transparency changes. Br J Ophthalmol.

[11] Malhotra A., Minja F.J., Crum A., Burrowes D. (2011). Ocular anatomy and cross-sectional imaging of the eye. Semin Ultrasound CT MR.

[12] Augusteyn R.C., Stevens A. (1998). Macromolecular structure of the eye lens: Polymer science and the eye. Prog Polym Sci.

[13] Mathias R.T., Rae J.L., Baldo G.J. (1997). Physiological properties of the normal lens. Physiol Rev.

[14] Thariat J., Racadot S., Pointreau Y. (2016). Intensity-modulated radiotherapy of head and neck cancers: Dose effects on the ocular, orbital and eyelid structures. Cancer Radiother.

[15] Minamoto A., Taniguchi H., Yoshitani N. (2004). Cataract in atomic bomb survivors. Int J Radiat Biol.

[16] Nakashima E., Neriishi K., Minamoto A. (2006). A reanalysis of atomic-bomb cataract data, 2000-2002: A threshold analysis. Health Phys.

[17] Cogan D.G., Martin S.F., Kimura S.J. (1949). Atom bomb cataracts. Science.

[18] Kimura S.J., Ikui H. (1951). Atomic-bomb radiation cataract. Case report with histopathologic study. Am J Ophthalmol.

[19] Otake M., Finch S.C., Choshi K., Takaku I., Mishima H., Takase T. (1992). Radiation-related ophthalmological changes and aging among Hiroshima and Nagasaki A-bomb survivors: A reanalysis. Radiat Res.

[20] Neriishi K., Nakashima E., Minamoto A. (2007). Postoperative cataract cases among atomic bomb survivors: Radiation dose response and threshold. Radiat Res.

[21] Day R., Gorin M.B., Eller A.W. (1995). Prevalence of lens changes in Ukrainian children residing around Chernobyl. Health Phys.

[22] Worgul B.V., Kundiyev Y.I., Sergiyenko N.M. (2007). Cataracts among Chernobyl clean-up workers: Implications regarding permissible eye exposures. Radiat Res.

[23] Chen W.L., Hwang J.S., Hu T.H., Chen M.S., Chang W.P. (2001). Lenticular opacities in populations exposed to chronic low-dose-rate gamma radiation from radiocontaminated buildings in Taiwan. Radiat Res.

[24] Su Y., Wang Y., Yoshinaga S. (2021). Lens opacity prevalence among the residents in high natural background radiation area in Yangjiang, China. J Radiat Res.

[25] Karatasakis A., Brilakis H.S., Danek B.A. (2018). Radiation-associated lens changes in the cardiac catheterization laboratory: Results from the IC-CATARACT (CATaracts Attributed to RAdiation in the CaTh lab) study. Catheter Cardiovasc Interv.

[26] Scheidemann-Wesp U., Gianicolo E.A.L., Cámara R.J. (2019). Ionising radiation and lens opacities in interventional physicians: Results of a German pilot study. J Radiol Prot.

[27] Bitarafan Rajabi A., Noohi F., Hashemi H. (2015). Ionizing radiation-induced cataract in interventional cardiology staff. Res Cardiovasc Med.

[28] Chodick G., Bekiroglu N., Hauptmann M. (2008). Risk of cataract after exposure to low doses of ionizing radiation: A 20-year prospective cohort study among US radiologic technologists. Am J Epidemiol.

[29] Chylack L.T., Peterson L.E., Feiveson A.H. (2009). NASA study of cataract in astronauts (NASCA). Report 1: Cross-sectional study of the relationship of exposure to space radiation and risk of lens opacity. Radiat Res.

[30] Little M.P., Kitahara C.M., Cahoon E.K. (2018). Occupational radiation exposure and risk of cataract incidence in a cohort of US radiologic technologists. Eur J Epidemiol.

[31] Auvinen A., Kivelä T., Heinävaara S., Mrena S. (Aug 2015). Eye lens opacities among physicians occupationally exposed to ionizing radiation. Ann Occup Hyg.

[32] Alhasan A.S., Aalam W.A. (2022). Eye lens opacities and cataracts among physicians and healthcare workers occupationally exposed to radiation: A systematic review and meta-analysis. Saudi Med J.

[33] Vano E., Kleiman N.J., Duran A., Romano-Miller M., Rehani M.M. (2013). Radiation-associated lens opacities in catheterization personnel: Results of a survey and direct assessments. J Vasc Interv Radiol.

[34] Vano E., Kleiman N.J., Duran A., Rehani M.M., Echeverri D., Cabrera M. (2010). Radiation cataract risk in interventional cardiology personnel. Radiat Res.

[35] Ciraj-Bjelac O., Rehani M.M., Sim K.H., Liew H.B., Vano E., Kleiman N.J. (2010). Risk for radiation-induced cataract for staff in interventional cardiology: Is there reason for concern?. Catheter Cardiovasc Interv.

[36] Rose A., Rae W.I.D., Sweetlove M.A., Ngetu L., Benadjaoud M.A., Marais W. (2022). Radiation induced cataracts in interventionalists occupationally exposed to ionising radiation. SA J Radiol.

[37] Jacob S., Boveda S., Bar O. (2013). Interventional cardiologists and risk of radiation-induced cataract: Results of a French multicenter observational study. Int J Cardiol.

[38] Barbosa A.H.P., Medeiros R.B., Corpa A.M.R. (2019). Prevalence of lens opacity in interventional cardiologists and professional working in the hemodynamics in Brazil. Arq Bras Cardiol.

[39] Azizova T.V., Hamada N., Bragin E.V., Bannikova M.V., Grigoryeva E.S. (2019). Risk of cataract removal surgery in Mayak PA workers occupationally exposed to ionizing radiation over prolonged periods. Radiat Environ Biophys.

[40] Rastegar N., Eckart P., Mertz M. (2002). Radiation-induced cataract in astronauts and cosmonauts. Graefes Arch Clin Exp Ophthalmol.

[41] Facius R. (2006). No evidence for the causation by cosmic radiation of nuclear cataracts in pilots. Arch Ophthalmol.

[42] Klein B.E., Klein R., Linton K.L., Franke T. (1993). Diagnostic x-ray exposure and lens opacities: The Beaver Dam Eye Study. Am J Public Health.

[43] Emami P., Gaudreau K., Little M.P. (2024). Assessment of cataract risk after diagnostic head CT scan radiation exposure in Ontario, Canada. Radiat Res.

[44] Gaudreau K., Thome C., Weaver B., Boreham D.R. (2020). Cataract formation and low-dose radiation exposure from head Computed Tomography (CT) scans in Ontario, Canada, 1994-2015. Radiat Res.

[45] Thariat J., Jacob S., Caujolle J.P. (2017). Cataract avoidance with proton therapy in ocular melanomas. Invest Ophthalmol Vis Sci.

[46] Mathis T., Rosier L., Meniai F. (2019). The lens opacities classification system III grading in irradiated uveal melanomas to characterize proton therapy-induced cataracts. Am J Ophthalmol.

[47] Uwineza A., Kalligeraki A.A., Hamada N., Jarrin M., Quinlan R.A. (2019). Cataractogenic load - A concept to study the contribution of ionizing radiation to accelerated aging in the eye lens. Mutat Res Rev Mutat Res.

[48] Fukutsu K., Kase S., Ishijima K., Kinoshita R., Ishida S. (2018). The clinical features of radiation cataract in patients with ocular adnexal mucosa-associated lymphoid tissue lymphoma. Radiat Oncol.

[49] Bayraktar Ş, Tuncer S., Özgün C., Peksayar G., Kebudi R. (2018). Surgical outcomes in radiation-induced cataracts after external-beam radiotherapy in retinoblastoma. Turk J Ophthalmol.

[50] Nguyen S.M., Sison J., Jones M. (2019). Lens dose-response prediction modeling and cataract incidence in patients with retinoblastoma after lens-sparing or whole-eye radiation therapy. Int J Radiat Oncol Biol Phys.

[51] Chodick G., Sigurdson A.J., Kleinerman R.A. (2016). The risk of cataract among survivors of childhood and adolescent cancer: A report from the childhood cancer survivor study. Radiat Res.

[52] Allodji R.S., Diallo I., El-Fayech C. (2016). Association of radiation dose to the eyes with the risk for cataract after nonretinoblastoma solid cancers in childhood. JAMA Ophthalmol.

[53] van Kempen-Harteveld M.L., Struikmans H., Kal H.B. (2002). Cataract after total body irradiation and bone marrow transplantation: Degree of visual impairment. Int J Radiat Oncol Biol Phys.

[54] van Kempen-Harteveld M.L., van Weel-Sipman M.H., Emmens C. (2003). Eye shielding during total body irradiation for bone marrow transplantation in children transplanted for a hematological disorder: Risks and benefits. Bone Marrow Transplant.

[55] Horwitz M., Auquier P., Barlogis V. (2015). Incidence and risk factors for cataract after haematopoietic stem cell transplantation for childhood leukaemia: An LEA study. Br J Haematol.

[56] Chodick G., Kleinerman R.A., Stovall M. (2009). Risk of cataract extraction among adult retinoblastoma survivors. Arch Ophthalmol.

[57] Collaborative Ocular Melanoma Study Group (2007). Incidence of cataract and outcomes after cataract surgery in the first 5 years after iodine 125 brachytherapy in the Collaborative Ocular Melanoma Study: COMS Report No. 27. Ophthalmology.

[58] Gragoudas E.S., Egan K.M., Arrigg P.G., Seddon J.M., Glynn R.J., Munzenrider J.E. (1992). Cataract extraction after proton beam irradiation for malignant melanoma of the eye. Arch Ophthalmol.

[59] Ebrahimi-Khankook A., Vejdani-Noghreiyan A. (2018). Dosimetric comparison between realistic ocular model and other models for COMS plaque brachytherapy with (103)Pd, (131)Cs, and (125)I radioisotopes. Radiat Environ Biophys.

[60] Puusaari I., Heikkonen J., Kivelä T. (2004). Effect of radiation dose on ocular complications after iodine brachytherapy for large uveal melanoma: Empirical data and simulation of collimating plaques. Invest Ophthalmol Vis Sci.

[61] Finger P.T. (2000). Tumour location affects the incidence of cataract and retinopathy after ophthalmic plaque radiation therapy. Br J Ophthalmol.

[62] Finger P.T., Chin K.J., Yu G.P., Patel N.S. (2011). Risk factors for cataract after palladium-103 ophthalmic plaque radiation therapy. Int J Radiat Oncol Biol Phys.

[63] Lin D., Lapen K., Sherer M.V. (2020). A systematic review of contouring guidelines in radiation oncology: Analysis of frequency, methodology, and delivery of consensus recommendations. Int J Radiat Oncol Biol Phys.

[64] Hoeben B.A.W., Seravalli E., Wood A.M.L. (2021). Influence of eye movement on lens dose and optic nerve target coverage during craniospinal irradiation. Clin Transl Radiat Oncol.

[65] Ding S., Liu H., Zhang L. (2023). Influence of eyes movement on lens dose during MR-guided radiotherapy for brain tumor. Int J Radiat Oncol Biol Phys.

[66] Piotrowski T., Ryczkowski A., Adamczyk M., Jodda A. (2015). Estimation of the planning organ at risk volume for the lenses during radiation therapy for nasal cavity and paranasal sinus cancer. J Med Imaging Radiat Oncol.

[67] Geneser S.E., Chang J.S., Chen J., Yom S.S., Garsa A.A. (2015). Expansions for planning target volume (PTV) and planning risk volume (PRV) to account for optic nerve and lens motion. Int J Radiat Oncol Biol Phys.

[68] Jaywant S.M., Osei E.K., Ladak S. (2003). Stereotactic radiotherapy in the treatment of ocular melanoma: A noninvasive eye fixation aid and tracking system. J Appl Clin Med Phys.

[69] Qi H., Hu L., Huang S. (2025). Proton therapy for uveal melanoma on a pencil beam scanning gantry. Adv Radiat Oncol.

[70] Graessl A., Muhle M., Schwerter M. (2014). Ophthalmic magnetic resonance imaging at 7 T using a 6-channel transceiver radiofrequency coil array in healthy subjects and patients with intraocular masses. Invest Radiol.

[71] Nguyen H.G., Sznitman R., Maeder P. (2018). Personalized anatomic eye model from T1-weighted volume interpolated gradient echo magnetic resonance imaging of patients with uveal melanoma. Int J Radiat Oncol Biol Phys.

[72] Fleury E., Trnková P., Erdal E. (2021). Three-dimensional MRI-based treatment planning approach for non-invasive ocular proton therapy. Med Phys.

[73] Ruan X., Liu Z., Luo L., Liu Y. (2020). The structure of the lens and its associations with the visual quality. BMJ Open Ophthalmol.

[74] Huang K., Rhee D.J., Ger R. (2021). Impact of slice thickness, pixel size, and CT dose on the performance of automatic contouring algorithms. J Appl Clin Med Phys.

[75] Yeltokova M., Zharliganova D., Shaidarov M. (2015). Deterministic effect of lens at leukergy of patients who received low doses of ionising radiation. Radiat Prot Dosimetry.

[76] Arefpour A.M., Bahrami M., Haghparast A., Khoshgard K., Aryaei Tabar H., Farshchian N. (2021). Evaluating dose-response of cataract induction in radiotherapy of head and neck cancers patients. J Biomed Phys Eng.

[77] Hall P., Granath F., Lundell M., Olsson K., Holm L.E. (1999). Lenticular opacities in individuals exposed to ionizing radiation in infancy. Radiat Res.

[78] Wilde G., Sjöstrand J. (1997). A clinical study of radiation cataract formation in adult life following gamma irradiation of the lens in early childhood. Br J Ophthalmol.

[79] Belkacémi Y., Ozsahin M., Pène F. (1996). Cataractogenesis after total body irradiation. Int J Radiat Oncol Biol Phys.

